# Emerging Roles of FGFR3 and FGF23-Associated Signaling in Renal Fibrosis: Molecular Integration, Clinical Correlates, and Therapeutic Perspectives

**DOI:** 10.3390/biom16101431

**Published:** 2026-10-02

**Authors:** Ying Fu, Hengping Wang, Lei Wang, Huiyan Wang

**Affiliations:** 1 Jilin Medical University, Jilin 132013, China; 2 National Engineering Research Center of Cell Growth Factor Drugs and Protein Preparations (Jilin Center), Jilin 132013, China; 3 Jilin Jinuo Biological Engineering Co., Ltd., Changchun 130600, China

**Keywords:** FGFR3, FGF23, Klotho, renal fibrosis, chronic kidney disease, FGF signaling, antifibrotic therapy

## Abstract

Renal fibrosis represents the final common pathway of chronic kidney disease (CKD) progression and remains a major therapeutic challenge. Fibroblast growth factor receptor 3 (FGFR3) has emerged as a context-dependent component of fibroblast growth factor (FGF) signaling, but its renal role remains incompletely defined because direct receptor-specific evidence is scarce. This review integrates kidney-specific FGFR3 studies with endocrine FGF23–Klotho biology and broader FGF/FGFR networks using an explicit evidence hierarchy. Current direct renal evidence is limited to two 2025 experimental studies: an FGF18-associated FGFR3–AMPK–NOX4 program that attenuated folic acid-induced fibrosis, and a single toxicant model reporting an FGF9–FGFR3–PI3K/Akt program associated with fibroblast proliferation. The latter requires independent replication before generalization beyond toxicant nephropathy. By contrast, physiological renal FGF23 signaling is best established through FGFR1c–α-Klotho, whereas the strongest evidence for Klotho-independent pathological FGF23 signaling involves FGFR4 in cardiovascular tissues; direct evidence that FGF23 promotes renal fibrosis specifically through FGFR3 is lacking. Accordingly, FGFR3 should be viewed as an emerging, ligand- and compartment-dependent signaling component rather than a validated CKD therapeutic target. Future priorities include human kidney spatial and phosphoproteomic profiling, cell-specific FGFR3 perturbation, longitudinal FGF23/Klotho biomarker studies, and mechanism-based evaluation of FGFR-directed strategies with integrated renal and mineral-metabolism endpoints.

## 1. Introduction

Chronic kidney disease (CKD) affects approximately one in ten adults worldwide and progresses, in many etiologies, through tubulointerstitial fibrosis—the strongest histological predictor of renal functional decline [1,2,3]. Despite decades of progress in blood pressure, glycemic, and renin–angiotensin–aldosterone system (RAAS) blockade, agents that directly halt or reverse renal scarring remain limited in clinical practice [1,4,5,6]. Identifying druggable signaling nodes that convert maladaptive repair into progressive fibrosis is therefore a central goal of nephrology research [2,5,7].

Fibroblast growth factor receptors (FGFR1–4) transduce paracrine and endocrine FGF signals that regulate development, epithelial repair, angiogenesis, metabolism, and tissue remodeling [8,9]. Recent work has further emphasized that FGF/FGFR signaling operates at the immune–stromal interface, coordinating inflammatory and tissue-resident stromal programs during chronic disease progression [10]. Among these receptors, FGFR3 is best known for gain-of-function mutations in skeletal dysplasias and driver alterations in urothelial carcinoma [11,12,13]. In extra-renal fibrosis, FGFR3 has been implicated in profibrotic signaling in systemic sclerosis and in experimental pulmonary fibroblast activation [14,15].

Renal fibrosis research occupies a peculiar position in this landscape. Nephrologists routinely measure FGF23 and discuss Klotho in clinic, yet rarely frame these hormones as FGFR3 biology. Conversely, oncology and skeletal genetics literature discusses FGFR3 mutations and inhibitors extensively, yet rarely addresses tubulointerstitial scarring [8,11,16]. This review integrates these bodies of literature through an evidence-graded framework, prioritizing kidney-direct FGFR3 experiments [17,18] and FGF23-axis renal data [19,20,21,22,23,24,25,26,27] over extrapolation from bladder cancer cell lines or chondrocyte genetics [12,13].

Whether FGFR3 plays a comparable role in human renal fibrosis remains unresolved. Kidney-specific evidence is limited to two experimental studies published in 2025 [17,18], and neither establishes a validated human therapeutic target. Accordingly, this review distinguishes direct renal receptor-specific evidence from renal but receptor-unspecified findings, human associations, extra-renal mechanistic studies, and oncology or skeletal extrapolation.

A recent review by Zhong et al. framed FGF/FGFR signaling as an immune–stromal regulator across autoimmune rheumatic diseases [10]. The present review addresses a different question: how kidney-specific FGFR3 evidence, local ligand context, and the endocrine FGF23–Klotho system can be integrated without equating indirect evidence with a renal FGF23–FGFR3 fibrotic mechanism.

This narrative, state-of-the-art review aims to: (i) summarize renal fibrosis biology relevant to receptor tyrosine kinase (RTK) signaling; (ii) critically evaluate direct renal FGFR3 evidence; (iii) separate established FGF23–FGFR1c–α-Klotho physiology and FGFR4-mediated non-canonical signaling from the hypothetical contribution of FGFR3 to FGF23-associated renal fibrosis; (iv) assess translational implications of FGF23 and FGFR-directed strategies; and (v) define evidence-based priorities for future research.

This article is a narrative, evidence-informed review rather than a systematic review or meta-analysis. The literature search was updated through 1 September 2026 using PubMed/MEDLINE, Web of Science, and Scopus with combinations of the terms FGFR3, FGFR1, FGFR2, FGFR4, FGF9, FGF18, FGF23, Klotho, renal fibrosis, kidney fibrosis, CKD, AKI, tubulointerstitial fibrosis, and receptor tyrosine kinase. English-language peer-reviewed primary studies, clinically relevant cohort studies, major mechanistic reviews, and key translational trials were considered; backward and forward citation tracking was used for pivotal studies. Retraction status and bibliographic details of contested or high-impact citations were checked against PubMed and publisher records. Evidence was categorized as Tier A, direct receptor-specific renal experimental evidence; Tier B, renal FGF/FGFR evidence without receptor-specific attribution; Tier C, human renal/CKD associative or biomarker evidence; Tier D, extra-renal mechanistic FGFR3 evidence; and Tier E, oncology/skeletal or therapeutic-safety extrapolation. These tiers indicate mechanistic proximity to renal FGFR3 biology and are not a quantitative risk-of-bias score (Table 1).

## 2. Pathophysiology of Renal Fibrosis: A Framework for RTK Signaling

Renal fibrosis is not a distinct disease entity but a convergent wound-healing response to glomerular, tubular, vascular, and interstitial injury [2,7]. This process typically evolves through overlapping phases of priming, activation, execution, and progression, characterized by persistent inflammation and defective resolution of extracellular matrix deposition [2]. Tubular epithelial cells are increasingly recognized as active contributors to fibrosis: following injury, they release cytokines, undergo partial epithelial–mesenchymal transition (EMT), and promote myofibroblast recruitment [39,40]. Interstitial myofibroblasts arise predominantly from resident pericytes and fibroblasts rather than through extensive EMT, although EMT-associated transcriptional programs remain biologically relevant [36,40].

TGF-β/Smad signaling is the best-characterized profibrotic pathway in CKD, regulating Smad3 activation, microRNA networks, and extracellular matrix (ECM) gene expression [7,41]. Parallel pathways, including PDGF, Wnt, Notch, and receptor tyrosine kinase signaling, further influence fibroblast survival, activation, and matrix synthesis [2,4]. FGF/FGFR signaling occupies a context-dependent position within this network: it contributes to nephrogenesis and adaptive tissue repair, yet sustained or dysregulated activation may promote fibroblast proliferation and excessive ECM deposition [28,29,42].

Clinically, the extent of tubulointerstitial fibrosis and tubular atrophy observed on kidney biopsy is an important histopathological predictor of CKD progression and kidney failure [1,43]. Antifibrotic therapeutic development has broadened to include SGLT2 inhibitors and nonsteroidal mineralocorticoid receptor antagonists, both of which have demonstrated kidney-protective effects in large clinical trials, as well as investigational receptor tyrosine kinase inhibitors evaluated primarily in preclinical fibrotic models [2,5,44,45,46]. However, no FGFR3-directed therapy has yet been evaluated in clinical trials specifically for CKD or renal fibrosis [16].

### 2.1. Cellular Compartments and Matrix Remodeling

Renal fibrosis drives progressive remodeling of the interstitium through excessive deposition of fibrillar collagens (particularly collagen I and III), fibronectin accumulation, and tubular basement membrane thickening, ultimately resulting in disruption of tissue architecture and progressive loss of renal function [2,7,40]. Pericytes detach from peritubular capillaries and represent a major source of matrix-producing myofibroblasts after injury, while resident fibroblasts undergo activation, proliferate, and secrete lysyl oxidase (LOX) family enzymes that promote collagen cross-linking and extracellular matrix (ECM) stabilization [40,47,48]. Macrophages actively regulate fibrotic progression by releasing TGF-β, PDGF, and inflammatory mediators, thereby sustaining fibroblast activation and modulating tubular injury responses, including local growth factor signaling networks [7,39,49]. In parallel, endothelial dysfunction exacerbates tissue hypoxia, vascular rarefaction, and inflammatory cell recruitment, generating a profibrotic microenvironment in which receptor tyrosine kinase (RTK) signaling, including FGF/FGFR pathways, may contribute to the transition from adaptive repair toward maladaptive fibrotic remodeling [30,50,51].

Tubular injury biomarkers, such as kidney injury molecule-1 (KIM-1) and neutrophil gelatinase-associated lipocalin (NGAL), together with circulating FGF23 levels, illustrate the potential divergence between parenchymal injury signals and endocrine responses during CKD progression [19,52,53]. For example, circulating FGF23 may rise early during CKD development before substantial structural fibrosis becomes evident, whereas established interstitial fibrosis may persist despite changes in circulating biomarkers [19,21,26,27,52]. Therefore, integrating histopathological fibrosis assessment with endocrine profiling and pathway-specific molecular analyses will be essential to determine whether FGFR3 pathway activation correlates with renal scarring independently of estimated glomerular filtration rate (eGFR) [1,5]. Such multidimensional approaches may help define biomarker signatures for identifying CKD subgroups with distinct FGF/FGFR signaling states and therapeutic responsiveness.

### 2.2. Why Receptor Tyrosine Kinases Matter in Antifibrotic Design

Unlike cytokine-neutralizing antibodies that typically inhibit individual ligand–receptor interactions, receptor tyrosine kinase (RTK) inhibitors can modulate downstream signaling hubs that integrate multiple extracellular cues and regulate complex transcriptional programs [4,54]. FGFR family members are particularly attractive therapeutic candidates because they function at the interface of developmental morphogen signaling, metabolic regulation, and adult tissue repair processes [8,9,55]. However, substantial functional redundancy among FGFR1–4, together with ligand-specific requirements for co-receptors such as α-Klotho and heparan sulfate proteoglycans, indicates that pan-FGFR inhibition, isoform-selective blockade, and ligand-targeted modulation may produce distinct biological outcomes [16,20,56]. Therefore, therapeutic interpretation of FGFR signaling requires careful consideration of receptor subtype, ligand availability, cellular compartment, and disease context. In this review, FGFR3 biology is emphasized only where supported by direct renal evidence or FGF23-axis studies, whereas FGFR1/2-related findings are discussed as complementary evidence that provides broader context for FGF/FGFR signaling in renal fibrosis [30,31,32,33,34,35,50].

## 3. FGFR3 Molecular Biology and Signaling Logic

### 3.1. Structure, Activation, and Canonical Pathways

FGFR3 is a single-pass transmembrane receptor tyrosine kinase composed of three extracellular immunoglobulin-like domains, a transmembrane helix, and an intracellular kinase domain [11,35]. Ligand engagement by FGFs, including FGF1, FGF9, FGF18, and FGF23 in a context-dependent manner, promotes receptor dimerization and autophosphorylation, leading to the recruitment of adaptor proteins and activation of downstream signaling cascades, including the RAS–MAPK/ERK and PI3K–AKT pathways [11,35,57]. The specificity and biological consequences of FGFR3 signaling are further determined by extracellular cofactors and tissue-specific co-receptors. Heparan sulfate proteoglycans stabilize ligand–receptor complexes and regulate FGF signaling gradients, whereas α-Klotho functions as an essential co-receptor for endocrine FGF23 signaling and defines tissue responsiveness to circulating FGF23 [27,56,58,59,60].

Gain-of-function mutations in FGFR3 cause achondroplasia and related skeletal dysplasias through constitutive activation of MAPK signaling in growth plate chondrocytes, demonstrating the developmental consequences of sustained FGFR3 activity [11,61]. In oncology, activating FGFR3 mutations and fusions define molecular subsets of urothelial carcinoma that may respond to FGFR-directed therapies [11,16,62]. In contrast, renal fibrosis is not characterized by recurrent FGFR3 genomic alterations but rather by context-dependent changes in receptor expression, ligand availability, and downstream pathway activity. Notably, aristolochic acid (AA)-associated nephropathy and upper tract urothelial carcinoma represent related exposure-associated disease spectra in which chronic tubular injury, fibrosis, and FGFR3-related genomic alterations may occur in distinct but biologically connected contexts [12,13,63].

### 3.2. FGFR3 in the FGF Receptor Family

The four FGFR paralogs exhibit distinct ligand-binding preferences, splice-isoform profiles, and cell-type-specific expression patterns, resulting in partially overlapping but non-equivalent functions during kidney injury. In experimental renal disease:

1. FGFR1 is involved in endothelial and tubular signaling during renal fibrogenesis. Angiotensin II–associated FGFR1 activation and endothelial FGFR1-related pathways have been linked to profibrotic remodeling, whereas apelin–FGFR1 interaction may exert protective effects by suppressing TGF-β-induced endothelial-to-mesenchymal transition [30,31,32,33,50].
2. FGFR2 expressed in interstitial fibroblasts contributes to post-ischemic fibrogenesis. Fibroblast-specific deletion of FGFR2 reduces fibroblast activation, extracellular matrix accumulation, and renal dysfunction following ischemia–reperfusion injury [34,35,64].
3. Kidney-specific evidence for FGFR3 remains comparatively limited and ligand-dependent. Direct studies implicate FGF18–FGFR3 signaling in an antifibrotic AMPK–NOX4 response and FGF9–FGFR3 signaling in profibrotic PI3K/Akt activation [17,18]. FGFR3 has also been implicated in FGF23-related signaling in extra-renal tissues, including bone and vasculature, and in TGF-β-driven profibrotic programs in systemic sclerosis [14,17,18,20,22,25,26,36].
4. FGFR4 is a well-established mediator of non-canonical, α-Klotho-independent FGF23 signaling, particularly in cardiac and other extra-renal tissues exposed to markedly elevated FGF23 concentrations in CKD [8,22,65].

Accordingly, FGFR3 should be interpreted within the broader FGFR family network. Broad multikinase inhibition cannot be used to infer an FGFR3-specific renal effect, and evidence from a retracted nintedanib renal-fibrosis report was excluded from the present synthesis. The remaining non-retracted renal nintedanib literature addresses other kidney disease contexts and is treated only as indirect pharmacological background [66].

### 3.3. Co-Receptor Logic and Spatial Regulation of FGFR3

FGFR3 ligand engagement is determined not only by receptor abundance but also by ligand availability, receptor splice isoforms, extracellular cofactors, and the spatial distribution of co-receptors. Cell-surface heparan sulfate proteoglycans facilitate the formation and stabilization of FGF–FGFR signaling complexes and regulate the local distribution and activity of paracrine FGFs [11,55,56,67]. Endocrine FGF23 signaling differs from that of canonical FGFs because it requires α-Klotho to form a high-affinity signaling complex, predominantly with FGFR1c in the kidney [20,27,68,69]. Although FGF23 ultimately regulates phosphate transport and vitamin D metabolism in proximal tubules, the precise nephron segments and intercellular mechanisms responsible for these effects remain incompletely resolved; therefore, α-Klotho–FGFR1c signaling should not be assigned exclusively to proximal tubular cells [24,70]. During CKD progression, renal membrane-bound and soluble α-Klotho levels decline, whereas circulating FGF23 concentrations increase markedly [21,22,71]. Under these conditions, very high FGF23 levels may activate lower-affinity, α-Klotho-independent pathways involving FGFR3 or FGFR4 in susceptible extra-renal and possibly renal cell types [20,23,65]. This altered receptor and co-receptor environment may help explain how FGF23 shifts from a physiological regulator of phosphate and vitamin D homeostasis toward a mediator of pathological signaling, particularly FGFR4–calcineurin–NFAT-dependent cardiac hypertrophy [20,65,72]. Experimental evidence links excessive FGF23 to renal fibrosis, whereas FGFR3 has been detected together with FGF23 in human vascular tissue associated with impaired kidney function [19,25]. However, direct evidence that a Klotho-dependent shift toward FGFR3/FGFR4 signaling contributes to human renal fibrosis remains lacking.

Spatially, FGFR3 signaling is expected to exhibit compartment-specific regulation. Direct renal studies indicate that FGF18–FGFR3 and FGF9–FGFR3 pathways exert distinct effects in tubular and tubulointerstitial compartments through AMPK- and PI3K/Akt-dependent mechanisms, respectively [17,18]. Evidence from extra-renal tissues further suggests that FGFR3 participates in FGF23-related signaling networks, although its functional contribution varies according to receptor context and co-receptor availability [20,26,36]. Current single-cell and spatial transcriptomic atlases of human CKD kidneys have defined major fibrotic cell populations but do not capture receptor activation states, highlighting the need for phospho-proteomic and spatial functional analyses to determine when and where FGFR3 signaling contributes to renal fibrosis [37,38].

### 3.4. FGFR3 in Oncology Versus Kidney: Cautionary Parallels

Oncology has established FGFR3 as a therapeutically actionable driver in molecularly defined subsets of urothelial carcinoma, with the FGFR inhibitor erdafitinib approved for metastatic urothelial cancers harboring susceptible FGFR2/3 alterations [8,58]. However, oncogenic FGFR3 activation through mutations, fusions, or overexpression differs fundamentally from the ligand-dependent and microenvironment-regulated FGFR signaling proposed in renal fibrosis. Despite these differences, convergence on shared downstream pathways, including MAPK and PI3K/Akt signaling, provides a mechanistic rationale for exploring FGFR-directed strategies beyond cancer biology [16,55]. The clinical overlap between urothelial malignancy and renal injury is further illustrated by aristolochic acid (AA)-associated nephropathy, in which chronic tubulointerstitial fibrosis and upper tract urothelial carcinoma may occur within the same exposure-related disease spectrum [12,13,63]. Importantly, FGFR3 genomic alterations in urothelial tumor cells should not be interpreted as evidence of FGFR3-driven renal fibrosis, because tumor-associated genetic activation and interstitial receptor signaling represent distinct biological processes. Therefore, reviews of renal FGFR3 biology must carefully distinguish oncogenic FGFR3 alterations from ligand-, receptor-, and microenvironment-dependent signaling events in the fibrotic kidney to avoid inappropriate therapeutic extrapolation.

## 4. Direct Evidence: FGFR3 in Renal Fibrosis

### 4.1. Antifibrotic FGF18–FGFR3–AMPK Axis

Zhou et al. provided the first direct evidence linking FGFR3 signaling to renal antifibrotic responses by demonstrating that FGF18 expression is increased in folic acid (FA)-induced murine renal fibrosis and in TGF-β-stimulated human proximal tubular epithelial (HK-2) cells [17]. Kidney-targeted FGF18 overexpression attenuated FA-induced renal fibrosis, reduced oxidative stress, and decreased fibrosis-associated marker expression. Mechanistically, the protective response was associated with FGFR3-linked enhancement of AMPK phosphorylation, suppression of NOX4-mediated reactive oxygen species (ROS) generation, and reduced fibrotic remodeling [17]. These findings identify FGFR3 as a potential mediator of protective FGF signaling in tubular injury and expand the conventional view of FGFR biology beyond its predominant association with oncogenic activation and fibrosis-promoting pathways [11,55].

Several aspects of this study are important for interpretation and future experimental design. First, the protective phenotype was primarily observed in tubular compartments and was closely associated with metabolic stress regulation and oxidative injury, suggesting that FGFR3–AMPK signaling may be particularly relevant in renal injuries characterized by prominent NOX4 activation [17,30]. Second, the study relied on FGF18 overexpression rather than selective FGFR3 activation or receptor-specific genetic manipulation; therefore, the relative contribution of FGFR3 compared with other potential FGF18 receptors remains to be fully defined [11,17,55]. Third, although HK-2 cell experiments provide mechanistic support for tubular responses, they cannot substitute for validation in human kidney tissue. Nevertheless, this study currently represents the strongest direct evidence that enhancement of a specific FGFR3-associated signaling program may exert antifibrotic effects in selected renal injury contexts, highlighting a therapeutic concept distinct from the FGFR inhibition strategies primarily developed in oncology [16,56].

### 4.2. Profibrotic FGF9–FGFR3–PI3K/Akt Axis in Toxic Nephropathy

Lyu et al. reported a profibrotic FGFR3-associated pathway in an environmental nephropathy model in which sodium p-perfluorous nonenoxybenzenesulfonate (OBS), a PFOS-related perfluoroalkyl substance (PFAS), induced renal fibrosis in mice [18]. Integrated metabolomic and molecular analyses linked Nα-acetyllysine depletion and lysyl oxidase activation to miR-140-5p suppression, increased FGF9/FGFR3 expression, PI3K/Akt activation, and fibroblast proliferation. Nα-acetyllysine supplementation, a miR-140-5p agomir, or LY294002 attenuated fibrosis and reduced pathway activation [18]. This single study supports a model-specific FGF9–FGFR3–PI3K/Akt mechanism in OBS nephrotoxicity, but independent replication, receptor-specific perturbation, and human validation are required before this pathway can be generalized to CKD.

The OBS study also provides mechanistic insights into environmental nephrology by illustrating how xenobiotic exposure may engage growth factor signaling networks during kidney injury. Epidemiological studies have linked PFAS exposure with altered kidney function and CKD-related outcomes, although the molecular mechanisms connecting environmental contaminants with renal fibrogenesis remain incompletely understood [73,74]. The identification of a lysine metabolism–miR-140-5p–FGF9/FGFR3 axis provides a potential framework for understanding how environmental stressors may regulate RTK-dependent fibrotic responses distinct from metabolic or ischemic kidney injury [2,18]. Whether similar FGFR3 pathway activation occurs in human PFAS-associated CKD remains unknown and requires validation in human cohorts [18,36].

### 4.3. Synthesis of Direct Evidence

Current direct renal evidence consists of two model-specific studies with divergent outcomes. FGF18-associated FGFR3 signaling was linked to AMPK activation, reduced NOX4-mediated oxidative stress, and attenuation of folic acid-induced fibrosis, whereas one OBS toxicant model reported FGF9–FGFR3–PI3K/Akt signaling associated with fibroblast proliferation and fibrosis [17,18]. The latter study is not retracted, but its findings have not yet been independently replicated.

These studies support the concept that FGFR3 output can vary with ligand and injury context, but they do not justify classifying FGFR3 as uniformly protective or pathogenic. Both pathways require receptor-specific genetic validation and confirmation in additional renal models and human tissue.

Accordingly, therapeutic conclusions should remain provisional until cell type-specific FGFR3 perturbation, receptor phosphorylation mapping, and independent replication define when FGFR3 activation is causal, compensatory, or epiphenomenal.

### 4.4. Methodological Comparison and Translational Implications

The two direct renal studies differ in ligand, injury model, downstream pathway, and phenotypic direction. Zhou et al. linked FGF18-associated FGFR3 signaling to an AMPK–NOX4 protective response in folic acid injury, whereas Lyu et al. reported an FGF9–FGFR3–PI3K/Akt profibrotic response in OBS nephrotoxicity [17,18]. Importantly, neither study used a cell type-specific FGFR3 knockout, and the FGF9 study currently represents a single toxicant model. Thus, the apparent ligand-dependent duality is a working model that requires independent replication rather than a basis for indiscriminate FGFR3 inhibition.

The relevance of current FGFR3 findings to human kidney disease remains indirect. Available renal studies are derived primarily from experimental injury models, whereas most clinical CKD arises from diabetic, hypertensive, ischemic, immune-mediated, and aging-related mechanisms [4,39]. Translating these observations into human disease will require validation in well-characterized kidney biopsy cohorts using standardized assessment of FGFR3 expression, phosphorylation status, ligand abundance, and spatial distribution of downstream signaling activity. Approaches such as phospho-specific immunostaining, imaging mass cytometry, and spatial proteomics may help determine whether ligand-specific FGFR3 activation correlates with fibrosis severity, disease etiology, or clinical progression independently of eGFR [1,5].

## 5. FGF23–Klotho–FGFR Signaling: A Major Clinical Link and an Emerging FGFR3 Hypothesis

Viewpoint: FGF23 provides the strongest clinical connection between FGF biology and CKD, but this should not be equated with an established renal FGF23–FGFR3 fibrotic axis. Physiological renal FGF23 signaling is best established through FGFR1c–α-Klotho, and the strongest Klotho-independent pathological evidence involves FGFR4 in cardiovascular tissues. Direct renal FGFR3 evidence remains limited to the ligand-specific FGF18 and FGF9 experimental models [17,18].

### 5.1. Endocrine FGF23 Physiology and Receptor Utilization

FGF23 is produced predominantly by osteocytes and osteoblasts and functions as an endocrine regulator of phosphate and vitamin D homeostasis [20,23,27]. Canonical renal signaling involves the formation of an FGF23–FGFR1c–α-Klotho complex, followed by activation of RAS–MAPK/ERK signaling and suppression of renal sodium–phosphate cotransporters and 1α-hydroxylase activity [20,27,69,75]. Although these physiological effects are expressed primarily in the proximal tubule, the precise nephron segments and intercellular mechanisms through which α-Klotho-dependent FGF23 signaling regulates proximal tubular phosphate handling remain incompletely resolved [76,77].

In advanced CKD, circulating FGF23 concentrations increase markedly as renal α-Klotho expression declines [21,22]. Under these conditions, FGF23 may activate lower-affinity, α-Klotho-independent FGFR pathways in susceptible tissues. The strongest mechanistic evidence supports FGFR4-mediated activation of PLCγ–calcineurin–NFAT signaling in cardiomyocytes, which promotes pathological cardiac hypertrophy [65,72]. FGFR3 has also been detected in FGF23-responsive vascular and skeletal tissues, but direct evidence that FGF23–FGFR3 signaling drives renal fibrosis remains limited [25,36]. Thus, the distinction between canonical FGFR1c–α-Klotho signaling and non-canonical FGFR signaling is important when considering FGF23 as both a biomarker of CKD progression and a potential mediator of systemic complications.

The best-established physiological function of FGF23 is the maintenance of phosphate balance through suppression of renal phosphate reabsorption and reduction in calcitriol synthesis, thereby coordinating communication among bone, kidney, and parathyroid tissues [20,23,27]. During early CKD, compensatory increases in FGF23 help preserve serum phosphate concentrations despite declining nephron function. As CKD progresses, however, persistent FGF23 elevation, Klotho deficiency, reduced calcitriol availability, and secondary hyperparathyroidism become interrelated components of CKD–mineral and bone disorder [21,78,79]. Elevated FGF23 is also strongly associated with cardiovascular morbidity and left ventricular hypertrophy, although the relative contributions of direct FGF23 signaling and accompanying disturbances in phosphate metabolism remain under investigation [65,72].

FGFR3 becomes relevant to this framework because altered ligand concentrations, co-receptor availability, and receptor expression may redistribute FGF23 signaling among FGFR subtypes in advanced disease. Experimental cardiorenal studies indicate that excessive FGF23 can aggravate renal fibrosis rather than serving solely as a marker of impaired kidney function [19]. Nevertheless, these studies have not established FGFR3 as the receptor responsible for this effect. Accordingly, therapeutic strategies that lower or modify FGF23 signaling should be evaluated not only for their effects on phosphate and vitamin D metabolism but also for cardiovascular safety and potential renal consequences [22,27]. Direct antifibrotic efficacy should remain an exploratory endpoint until receptor-specific mechanisms are established.

### 5.2. FGF23 Elevation Across CKD Stages and AKI–CKD Transition

FGF23 concentrations increase early during CKD progression, frequently preceding the development of overt hyperphosphatemia, and elevated circulating FGF23 levels are consistently associated with faster CKD progression, cardiovascular complications, and mortality [21,27,79]. This early rise reflects a compensatory response to declining nephron mass and impaired phosphate handling, but persistent FGF23 elevation may contribute to maladaptive signaling under conditions of advanced kidney disease.

**Figure 1:**
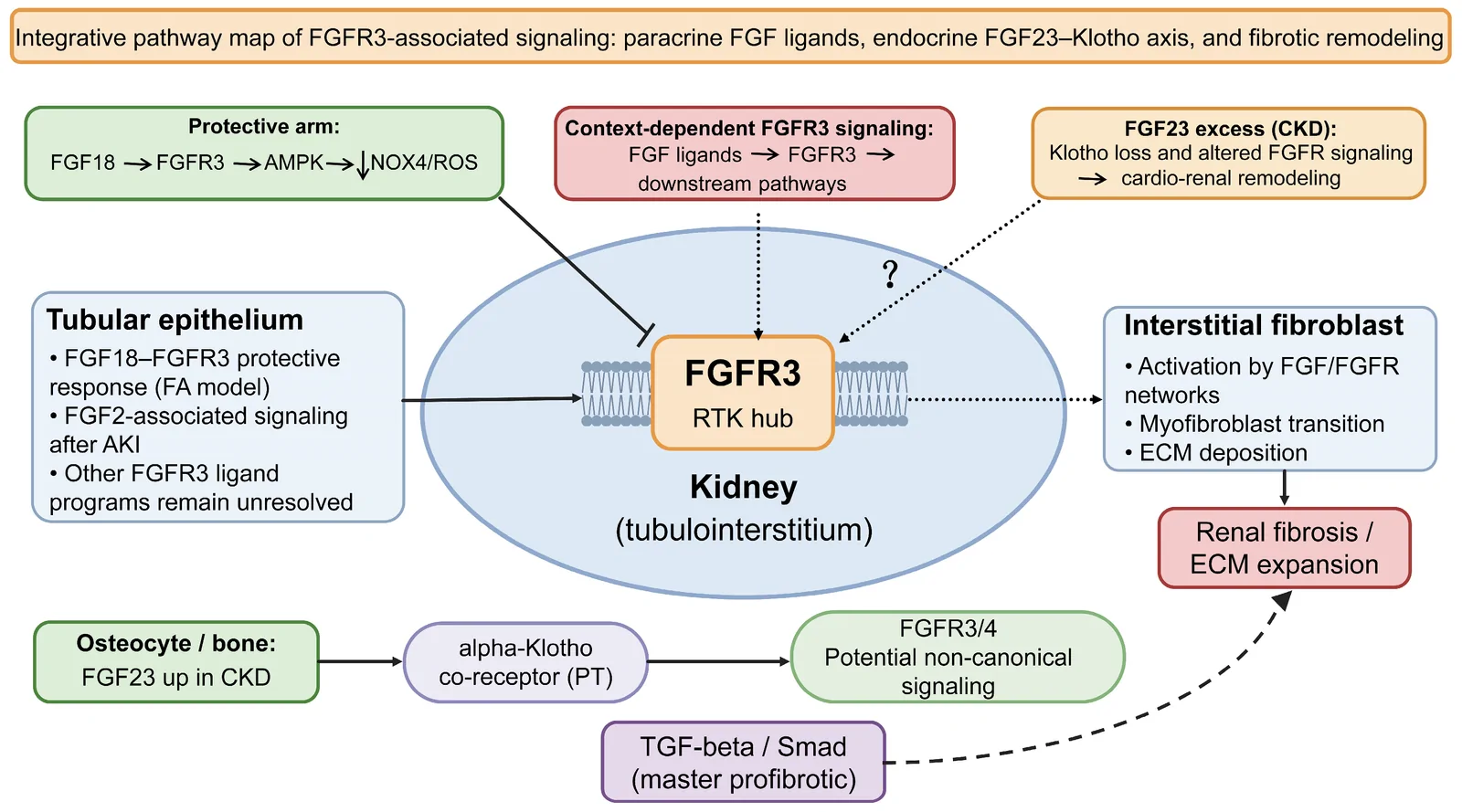
FGFR3-associated signaling networks in renal fibrosis: integration of paracrine FGF ligands and endocrine FGF23–Klotho signaling. Schematic evidence map distinguishing established, model-specific, and hypothetical relationships. Direct renal evidence includes FGF18-associated FGFR3–AMPK–NOX4 protection in folic acid injury and a single OBS toxicant study reporting FGF9–FGFR3–PI3K/Akt profibrotic signaling [17,18]. Physiological FGF23 signaling is primarily mediated by FGFR1c–α-Klotho, whereas FGFR4 has the strongest evidence for Klotho-independent pathological signaling [20,27,65,72]. A direct FGF23–FGFR3 renal fibrotic pathway has not been demonstrated; the corresponding link is therefore shown as a dashed, hypothesis-generating connection. Solid arrows denote experimentally supported relationships within the cited context, whereas dashed arrows denote indirect or unresolved links. All figure elements were created and assembled by the authors using WPS Presentation in WPS Office, Version 12.1.0.28505 (Kingsoft Office Software Corporation Limited, GuangZhou, China), proprietary software used under a valid license.

Declining renal α-Klotho expression accompanies CKD progression and alters the cellular context in which FGF23 signaling occurs [22,59,78]. Although the physiological relevance of a shift toward FGFR3/FGFR4 signaling in the fibrotic kidney remains incompletely established, reduced Klotho availability may modify FGFR receptor utilization and increase susceptibility to non-canonical FGF23 responses in selected tissues [20,23,36]. Clinical cohort studies further indicate that elevated FGF23 levels are associated with the transition from acute kidney injury (AKI) to CKD and with adverse long-term renal outcomes, even after adjustment for traditional markers of mineral metabolism [26]. These observations position FGF23 at the interface between acute tubular stress, chronic inflammation, and fibrotic remodeling, while highlighting the need to distinguish its role as a biomarker of disease severity from its potential contribution as a pathogenic mediator (Figure 1).

### 5.3. Direct Experimental Evidence: FGF23 Promotes Renal Fibrosis

Hao et al. provided direct experimental evidence that excessive FGF23 contributes to renal fibrotic remodeling in mice with type 2 cardiorenal syndrome [19]. In this model, elevated FGF23 levels were associated with increased extracellular matrix accumulation, fibroblast activation, and progression of renal fibrosis, supporting the concept that FGF23 may function as an active mediator of kidney injury rather than merely a biomarker reflecting reduced renal function or CKD severity [19]. Although the study did not define a specific FGFR subtype responsible for these effects, it demonstrates that excessive FGF23 signaling can promote a profibrotic renal environment under cardiometabolic stress.

Together with the detection of FGF23, FGFR1, and FGFR3 expression in coronary arterial tissues from individuals with impaired kidney function, these findings support a broader cardio–renal–skeletal FGF signaling framework in advanced CKD [36]. However, whether FGFR3 acts as a direct mediator of FGF23-associated renal fibrosis, a component of compensatory FGF signaling, or a marker of altered receptor responsiveness remains unresolved. Future studies using receptor-specific genetic manipulation, cell-type-resolved approaches, and spatial analysis of FGFR activation will be required to define the contribution of FGFR3 to FGF23-driven renal pathology.

### 5.4. FGF23/FGFR3 in Comorbidity: Bone, Transplant, and Vasculature

Beyond renal fibrosis, the FGF23–FGFR signaling axis contributes to multiple systemic complications relevant to CKD, including disturbances in skeletal growth, mineral metabolism, and vascular biology. In pediatric kidney transplant recipients, glucocorticoid exposure has been associated with increased FGF23 and FGFR3 expression in growth plate chondrocytes, and pharmacological FGFR3 inhibition partially rescues growth impairment, providing evidence for functional FGF23–FGFR3 interaction in the skeletal compartment under chronic kidney disease-related stress [25]. However, these findings should be interpreted primarily within the context of growth plate biology rather than as direct evidence for renal FGFR3-mediated fibrosis.

Therapeutic approaches targeting the FGF23–Klotho axis, including FGF23-neutralizing antibodies in selected hypophosphatemic disorders and experimental Klotho-restorative strategies, demonstrate that modulation of this pathway can alter mineral and endocrine phenotypes [22,27]. Nevertheless, whether such interventions provide renal antifibrotic benefits remains unknown. In CKD, therapeutic manipulation of FGF23 signaling requires careful consideration because FGF23 elevation may represent both an adaptive response to phosphate retention and a maladaptive driver of cardiovascular and tissue complications.

FGF23 has pleiotropic endocrine and Klotho-independent effects, whereas CKD-related inflammation and neurohormonal activation can further increase FGF23 concentrations [21,23]. These observations support systemic relevance of the pathway but do not establish an FGFR3-mediated renal fibrotic mechanism.

### 5.5. Integrating FGF23 with Paracrine FGFR3 Ligands (FGF9, FGF18)

FGF23 operates on a different spatial and temporal scale from paracrine FGF9 and FGF18, which act within local tissue microenvironments [17,18]. Figure 1 therefore presents endocrine FGF23 and local FGFR3 ligand programs as parallel, context-dependent components of the broader FGF network rather than as a proven common renal FGFR3 pathway.

1. Early/mild CKD: Circulating FGF23 increases before overt hyperphosphatemia develops, while renal α-Klotho expression gradually declines. During this phase, canonical FGF23–FGFR1c–Klotho signaling remains dominant and primarily maintains mineral homeostasis [20,27].
2. Progressive fibrotic remodeling: Local FGF18- and FGF9-associated FGFR3 programs have been reported in distinct experimental settings. FGF18-associated signaling was linked to AMPK–NOX4-mediated protection, whereas a single OBS toxicant study reported FGF9–PI3K/Akt-associated fibroblast activation [17,18]. These pathways should not be generalized beyond their respective models without independent validation.
3. Advanced CKD and cardiorenal stress: Marked FGF23 elevation and Klotho deficiency may alter the balance of FGFR signaling. Excess FGF23 can activate non-canonical FGFR pathways, with strongest evidence supporting FGFR4-mediated cardiovascular effects, whereas the contribution of FGFR3 to FGF23-associated renal fibrosis remains an unresolved hypothesis [19,36,65,72].

Therapeutically, these findings caution against nonspecific suppression of the FGF23–FGFR axis. Although reducing excessive FGF23 signaling may theoretically attenuate maladaptive remodeling in selected settings, systemic intervention may also disturb phosphate homeostasis and potentially interfere with protective paracrine FGF18–FGFR3 signaling [17,19]. Therefore, future FGF23- or FGFR-directed antifibrotic strategies will require ligand-, receptor-, and tissue-specific evaluation.

### 5.6. FGF23 as Biomarker, Effector, and Trial Covariate

In clinical nephrology, FGF23 is an established biomarker of CKD-mineral and bone disorder (CKD-MBD) and an independent predictor of cardiovascular risk and adverse renal outcomes [21,23,27]. Available assays include C-terminal and intact FGF23 measurements, which differ in analytical characteristics, biological interpretation, and susceptibility to changes in FGF23 cleavage; nevertheless, both measures frequently increase before the onset of overt hyperphosphatemia, supporting the utility of FGF23 as an early indicator of disturbed mineral metabolism during CKD progression [20,27,80]. Whether FGF23 directly drives renal fibrosis in humans remains unresolved. Experimental cardiorenal models demonstrate that excessive FGF23 can promote renal fibrotic remodeling, while clinical studies consistently show temporal association, biological gradient, and prognostic relationships between elevated FGF23 concentrations and CKD progression [18,21,26]. Thus, FGF23 currently occupies an intermediate position as both a biomarker of disease burden and a potential mediator of maladaptive signaling rather than a definitively established human fibrogenic factor. Serial FGF23 assessment after acute kidney injury (AKI) may help identify patients at increased risk of AKI-to-CKD transition and enrich future prevention trials targeting high-risk populations [9,26].

From a clinical trial-design perspective, FGF23 should be incorporated as a pharmacodynamic and safety covariate in future studies evaluating FGFR-directed therapies in CKD. Oncology experience with FGFR inhibitors, including erdafitinib, demonstrates that pathway inhibition can produce predictable mineral metabolism abnormalities, particularly hyperphosphatemia, reflecting interference with FGF–FGFR signaling [8,16,58]. Conversely, anti-FGF23 therapies are designed to restore phosphate handling in specific mineral disorders but require careful monitoring because excessive suppression of FGF23 may disrupt physiological bone–kidney endocrine communication [20,22].

Early-phase nephrology studies should integrate eGFR slope, quantitative fibrosis measures, and FGF23/Klotho trajectories so that functional, structural, and endocrine effects are assessed in parallel [16,19,36].

## 6. Paracrine FGF/FGFR Network in Kidney Injury and Fibrosis

Because FGFR3-specific renal evidence remains limited primarily to FGF9- and FGF18-dependent models, interpretation of FGFR3 biology requires integration with broader kidney-validated FGF/FGFR signaling networks. These studies demonstrate that individual FGFR paralogs operate within overlapping but non-equivalent pathways during tubular injury, fibroblast activation, and extracellular matrix remodeling.

### 6.1. Paracrine FGF2 Loops After AKI

Following ischemic or toxic acute kidney injury (AKI), persistent tubular stress and defective repair responses induce local FGF2 production, which promotes interstitial fibroblast activation and maladaptive fibrotic remodeling [28,29]. TGF-β stimulates FGF2 expression in injured tubular cells, whereas inhibition of autophagy-related signaling or blockade of FGF2 activity attenuates fibrosis in experimental models [28,29]. Although these studies primarily implicate FGFR1/FGFR2 signaling rather than FGFR3, they establish the broader principle that locally produced FGF ligands can function as paracrine mediators linking tubular injury to stromal activation. This framework provides important context for understanding how ligand-specific FGFR3 programs may emerge in distinct renal injury environments [17,18].

### 6.2. FGFR1 and FGFR2 in Chronic Fibrogenesis

Among FGFR family members, FGFR1 and FGFR2 have accumulated more extensive evidence in chronic renal fibrogenesis. Multiple studies implicate FGFR1 signaling in angiotensin II-mediated, endothelial, and metabolic kidney injury pathways [30,31,32,33,50]. Endothelial FGFR1 deficiency attenuates diabetic kidney fibrosis in experimental models, while FGFR1/YAP1 signaling has been proposed as a therapeutic target in endothelial maladaptive remodeling [30,50]. FGFR2 also contributes to stromal fibrogenesis. Fibroblast-specific FGFR2 deletion reduces post-ischemic matrix accumulation and fibroblast activation, indicating that FGFR2 signaling is required for efficient progression of interstitial fibrosis [64]. In addition, epigenetic alterations involving FGFR2 have been associated with age-related renal fibrotic remodeling [64]. Klotho, an important regulator of renal aging and mineral metabolism, suppresses tubulointerstitial fibrosis partly through inhibition of excessive FGF2-related signaling, linking endocrine aging pathways with local FGF activity [42].

Collectively, these findings indicate that FGFR1 and FGFR2 represent major contributors to established fibrogenic pathways in conventional CKD models, whereas FGFR3 appears to function within more restricted and context-dependent signaling environments. Current renal evidence supports a ligand-specific role for FGF18–FGFR3 signaling, while potential involvement of FGF23-related FGFR3 activation remains an emerging hypothesis requiring further validation [29]. This distinction is critical for therapeutic development, as pan-FGFR inhibition may suppress pathogenic signaling but may also interfere with protective FGFR-dependent repair programs. Future strategies should therefore prioritize isoform-selective, ligand-guided, and biomarker-informed approaches rather than nonspecific FGFR blockade [16].

### 6.3. Cross-Talk Among FGFR Paralogs and Implications for FGFR3 Inference

Because multikinase RTK inhibitors targeting FGFR pathways may attenuate experimental fibrosis, some interpretations have attempted to extrapolate these effects to FGFR3. However, without isoform-specific genetic or pharmacological validation, such attribution remains unsupported. In established CKD models, FGFR1 and FGFR2 have substantially stronger evidence for regulating endothelial responses, fibroblast activation, and extracellular matrix expansion in diabetic, hypertensive, and ischemic kidney injury [30,31,32,33,34,35,50]. In contrast, direct experimental evidence for FGFR3 involvement in renal fibrosis remains limited. Current renal data primarily support an FGF18–FGFR3-associated tubular protective response, whereas other proposed FGFR3 functions in kidney fibrosis remain incompletely validated [17,18].

Cross-talk among FGFR paralogs further complicates mechanistic interpretation. During AKI and chronic injury, increased local production of FGF ligands, including FGF2, may reshape the extracellular signaling environment and influence receptor engagement through differences in ligand availability, receptor affinity, alternative splicing, and co-receptor expression [8,11,28,29]. Although direct evidence demonstrating competitive ligand regulation of FGFR3 activity in fibrotic kidneys is lacking, this framework provides a potential explanation for why FGFR-directed interventions may produce different outcomes across heterogeneous kidney injury models.

Klotho deficiency represents an additional layer of complexity in FGF signaling regulation. Reduced Klotho expression during CKD enhances susceptibility to maladaptive FGF signaling, including excessive FGF2-associated fibrotic responses, while simultaneously altering the cellular context of FGF23 receptor utilization [42,59,78]. Therefore, Klotho restoration represents a potential upstream strategy to rebalance multiple FGF pathways rather than selectively targeting FGFR3 alone [22,27].

For preclinical prioritization, a ligand-context framework may improve experimental design: (i) in injury models characterized by FGF18 induction and tubular oxidative stress, enhancement of protective FGFR3-associated pathways or AMPK activation should be considered before receptor blockade [17]; (ii) in models suggesting FGF9/FGFR3 involvement, pathway activation should first be independently validated using receptor-specific genetic approaches before therapeutic inhibition is pursued; and (iii) in settings of excessive FGF23 and cardiorenal stress, FGF23 modulation should be evaluated together with receptor-specific signaling analysis and fibrosis endpoints [19]. This framework emphasizes that FGFR3 should be investigated as a context-dependent signaling node rather than assumed to be a universal profibrotic therapeutic target.

## 7. Integration with TGF-β, EMT, and Myofibroblast Biology

TGF-β/Smad signaling remains a central driver of renal fibroblast activation, extracellular matrix deposition, and maladaptive repair [7,41]. FGFR pathways intersect with this network in a context-dependent manner. In systemic sclerosis, TGF-β induces FGFR3/FGF9 and FGFR3 inhibition reduces dermal fibrosis, providing extra-renal mechanistic plausibility; in kidney, direct evidence is limited to the FGF18 and FGF9 experimental models [14,17,18]. Partial epithelial–mesenchymal transition contributes to tubular–stromal communication, but resident fibroblasts and pericytes are the major sources of renal myofibroblasts [39,40]. Thus, FGFR3 should be considered a potential modulator within the broader TGF-β-centered fibrotic network rather than a proven upstream driver.

## 8. Disease Contexts and Clinical Correlates

### 8.1. Diabetic and Metabolic CKD

Diabetic kidney disease combines hemodynamic, metabolic, oxidative, and inflammatory injury [39]. FGF23 is commonly elevated and associated with adverse cardiovascular outcomes, while experimental data support FGFR1-related endothelial and tubular fibrogenic signaling [21,23,42,50]. Direct FGFR3-specific evidence in diabetic kidney disease is lacking, so convergence of endocrine FGF23 and local FGFR3 pathways remains hypothetical.

### 8.2. Hypertension and RAAS Activation

In hypertensive kidney injury, angiotensin II activates FGFR1-related pathways in tubular and vascular compartments, whereas apelin–FGFR1 signaling can oppose TGF-β-induced endothelial-to-mesenchymal transition [31,32,33]. These findings support FGFR family cross-talk with RAAS-driven fibrosis but do not establish an FGFR3 role in hypertension.

### 8.3. Toxic and Environmental Nephropathy

Environmental nephropathies illustrate how exposure-specific injury may engage FGF signaling. Aristolochic acid causes chronic tubulointerstitial nephropathy and urothelial carcinoma, but tumor FGFR3 alterations should not be conflated with renal interstitial signaling [12,13]. Separately, the OBS study provides model-specific evidence for an FGF9–FGFR3–PI3K/Akt pathway [18]; replication in other toxicant models and human PFAS-associated CKD is needed.

### 8.4. AKI–CKD Transition

AKI-to-CKD transition is characterized by maladaptive repair and fibroblast activation [9,26]. FGF2 contributes to post-AKI stromal activation, and circulating FGF23 is associated with subsequent CKD risk [26,28,29]. Whether FGFR3 participates directly in this transition remains unknown and requires serial biomarker studies combined with spatial receptor-activation mapping.

### 8.5. CKD of Uncertain Etiology, Aging, and Immunologic Injury

Immune-mediated CKD, aging, and transplantation share tubulointerstitial fibrosis despite distinct triggers [2,7]. Disease-specific FGFR3 profiling is largely absent; available evidence instead supports conserved TGF-β/growth-factor activation, age-related FGF pathway changes, and altered FGF23/Klotho biology [14,21,27,35,41,59]. Etiology- and compartment-resolved studies are therefore required before FGFR3 can be assigned a therapeutic role in these settings.

**Figure 2:**
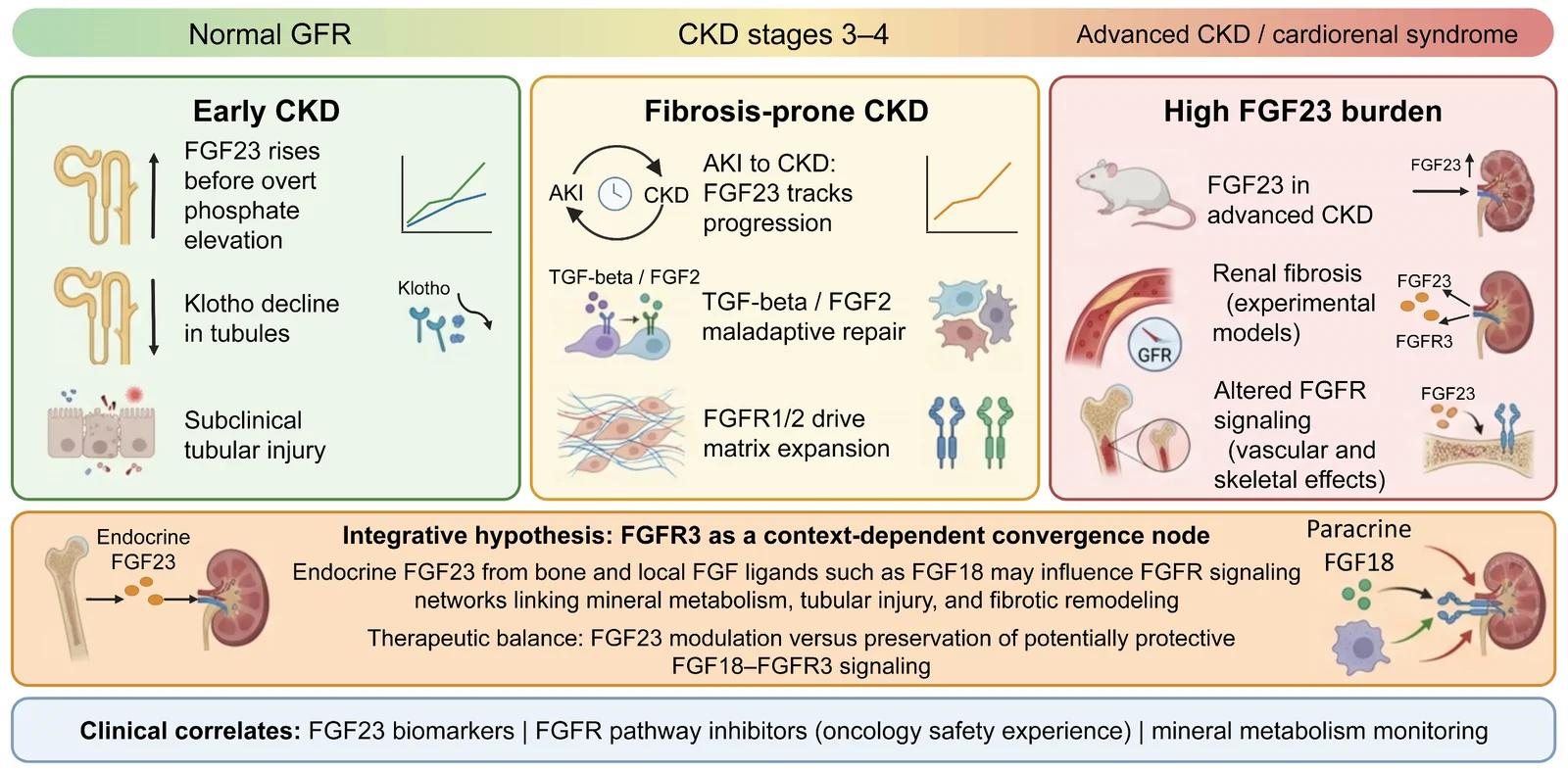
FGF23-associated FGFR signaling across CKD progression and renal fibrosis. Stage-based conceptual model linking early FGF23 elevation and α-Klotho decline [20,27,59] with maladaptive repair pathways involving TGF-β, FGF2, and FGFR1/2 [28,30,31,32,33,34,35,41,50]. Experimental cardiorenal data show that excessive FGF23 can accompany or promote renal fibrotic remodeling, whereas vascular FGFR3 detection and transplant-bone FGF23/FGFR3 signaling provide extra-renal contextual evidence [19,25,36]. These vascular, skeletal, and cardiorenal observations are not presented as proof of a tubulointerstitial FGF23–FGFR3 mechanism. The figure instead illustrates how endocrine FGF23 biology and local FGF programs may coexist during CKD progression, with FGFR3-specific renal causality remaining unresolved. All figure elements were created and assembled by the authors using WPS Presentation in WPS Office, Version 12.1.0.28505 (Kingsoft Office Software Corporation Limited, GuangZhou, China), proprietary software used under a valid license.

Figure 2 presents a stage-based integrative model linking CKD progression to changes in FGF23, Klotho, and FGFR signaling. Cardiorenal, vascular, and skeletal observations are included as indirect contextual evidence and are not intended to demonstrate a mechanistic route from FGF23 through FGFR3 to tubulointerstitial fibrosis. The model instead identifies testable interfaces that require receptor-specific renal validation [20,25,26,27,36].

## 9. Therapeutic Landscape: FGF23 Modulation and FGFR Inhibitors

### 9.1. FGF23-Targeted and Klotho-Restorative Strategies (Conceptual)

Anti-FGF23 therapies and Klotho-restorative approaches represent emerging strategies for modulating CKD-associated mineral and endocrine disturbances [19,22,27]. FGF23-neutralizing antibodies, such as burosumab, are established therapies for selected FGF23-mediated hypophosphatemic disorders but have not been evaluated as antifibrotic interventions in CKD. Extrapolation of FGF23 modulation toward renal fibrosis therefore requires caution: reducing excessive FGF23 signaling may attenuate cardiorenal remodeling in experimental models, but may also disrupt phosphate homeostasis and skeletal adaptation. Similarly, Klotho restoration may suppress maladaptive FGF signaling, including FGF2-associated fibrotic responses, while altering the balance of FGFR pathway activation in CKD [19,42,59,78]. To date, no clinical trial has evaluated FGF23-targeted therapy using renal fibrosis histology as a primary endpoint.

### 9.2. Broader FGFR-Directed Inhibition: Indirect Evidence and Limitations

Broad FGFR-containing multikinase pharmacology can inform pathway tractability but should not be treated as receptor-specific renal antifibrotic evidence. A previously cited 2017 nintedanib study reporting attenuation of renal fibrosis was retracted in 2026 and has therefore been removed from the evidence synthesis and tables. The remaining non-retracted kidney nintedanib study concerns autosomal-dominant polycystic kidney disease rather than renal fibrosis [66]. Clinical experience with nintedanib in systemic sclerosis-associated interstitial lung disease supports the tractability of FGFR-containing multikinase networks, while leaving the FGFR-specific contribution unresolved [10]. Accordingly, broad kinase-inhibitor experience is used here only as indirect pharmacological and safety context, not as evidence that FGFR3 is an antifibrotic kidney target.

### 9.3. Oncology FGFR3 Inhibitors

FGFR-directed therapies have achieved clinical validation in oncology. Erdafitinib, a selective FGFR inhibitor with activity against FGFR1–4, demonstrated clinical benefit in patients with metastatic urothelial carcinoma harboring susceptible FGFR alterations [8]. FGFR1–3-selective agents, including infigratinib, have further expanded understanding of FGFR biology, resistance mechanisms, and treatment-associated toxicities [16].

Oncology experience provides important safety considerations for potential nephrology applications. FGFR inhibitors commonly induce hyperphosphatemia due to interference with FGF23–FGFR1–Klotho physiology, together with dermatologic, nail, and ocular adverse events [16]. Because CKD patients already exhibit disturbed mineral metabolism and altered vascular calcification risk, chronic FGFR inhibition would require careful monitoring of phosphate balance, bone turnover, and cardiovascular consequences [21,27].

Although patients with upper tract urothelial carcinoma and CKD may receive FGFR inhibitors in clinical practice, available observations cannot determine whether FGFR modulation influences renal fibrosis progression. Therefore, repurposing oncology-derived FGFR3 inhibitors for CKD fibrosis remains hypothetical and requires biomarker-guided preclinical evaluation before clinical translation [4,5,16,17].

### 9.4. Isoform-Selective and Decoy Strategies

Isoform-selective approaches may provide advantages over broad FGFR inhibition by preserving physiological signaling functions. FGFR3 decoy receptors attenuated pulmonary myofibroblast transition in an extra-renal fibrosis model, whereas the renal FGF18 study suggests that some FGFR3-associated signaling may be protective in selected kidney injury contexts [15,17]. Together, these observations reinforce the need for ligand- and cell type-specific evaluation before FGFR3 blockade is considered in kidney disease.

FGFR3 phosphorylation has also been implicated in bladder and upper urinary tract remodeling, although these findings should be distinguished from renal interstitial fibrosis mechanisms [11].

### 9.5. Practical Roadmap for Therapeutic Development

Translating FGFR3/FGF23 biology into kidney trials requires three gated phases aligned with evidence tiers in this review:

Phase A—Biomarker enrichment (human observational): Prospective CKD and post-AKI cohorts with stored plasma and optional research biopsies should test whether FGFR pathway activity adds information beyond eGFR and mineral metabolism. Candidate measurements include circulating FGF23 and soluble Klotho; tissue FGFR3 and phospho-FGFR3; FGF18 and, where exposure context is relevant, FGF9; and pathway readouts such as phospho-AMPK/NOX4 and phospho-Akt [17,18,19,21,26]. These markers should be related to quantitative interstitial fibrosis and longitudinal renal outcomes.

Phase B—Mechanistic stratification: Compare FGF18-associated, FGF9-associated, FGF23-excess, and established FGFR1/2 renal models using cell-specific FGFR3 perturbation, fibrosis quantification, receptor phosphorylation, and matched downstream readouts [17,18,19,28,64]. FGFR3-selective approaches should be benchmarked against broader FGFR or FGF23-directed strategies with simultaneous renal and mineral-metabolism assessment [17,18,19].

Phase C—Clinical translation: Proceed only after biomarker enrichment and receptor-specific validation. Early CKD studies would require intensive phosphate, bone, and cardiovascular monitoring, while structural efficacy should be assessed with biopsy substudies or validated imaging surrogates where feasible [4,5,8,16,21,22].

Table 1 defines the evidence hierarchy used throughout this review. Table 2 summarizes representative experimental FGF/FGFR pathway evidence relevant to renal fibrosis, and Table 3 summarizes renal and cardio-renal evidence relevant to FGF23-associated FGFR signaling.

## 10. Critical Perspectives (Viewpoints)

Perspective 1—FGF23 is the strongest clinical bridge to altered FGF signaling in CKD, not proof of a renal FGF23–FGFR3 axis. FGF23 rises early, predicts adverse outcomes, and can promote renal fibrosis in a cardiorenal model, but receptor-specific renal causality remains unresolved [19,20,21,26,27].

Perspective 2—Ligand and injury context determine FGFR3 output. Direct renal data comprise an FGF18-associated protective program and one OBS toxicant study reporting an FGF9-associated profibrotic program [17,18]. Both are model-specific; the FGF9 study is not retracted but requires independent replication.

Perspective 3—Evidence tiering should govern mechanistic and therapeutic claims. Tier A renal receptor-specific studies are distinct from receptor-unspecified renal data, human associations, extra-renal mechanisms, and oncology/skeletal extrapolation (Table 1). Human kidney FGFR3 phosphorylation and cell-specific causality remain major gaps.

Perspective 4—Therapeutic claims must remain proportional to evidence. The retracted renal-fibrosis nintedanib study has been excluded; oncology and multikinase experience informs pharmacology and safety rather than CKD efficacy. FGFR3-selective intervention should not proceed clinically without biomarker enrichment and receptor-specific renal validation [10,16,17,58].

Perspective 5—Klotho restoration, FGF23-directed approaches, and human spatial profiling are complementary research strategies. These approaches may rebalance FGF signaling or identify biologically active FGFR states, but none currently validates FGFR3 as a CKD treatment target [21,22,27,59,78]. Figure 1 and Figure 2 therefore use evidence-aware conventions to distinguish established from hypothetical links.

## 11. Conclusions and Future Directions

FGFR3 and FGF23-associated signaling provide an emerging framework for studying renal fibrosis, but the evidence hierarchy is uneven. Direct renal FGFR3 evidence is limited to two model-specific studies; physiological FGF23 signaling is established through FGFR1c–α-Klotho, and FGFR4 has the strongest evidence for Klotho-independent pathological signaling. Experimental FGF23-driven renal fibrosis does not identify FGFR3 as the responsible receptor.

For translational researchers, we propose several priorities:

1. FGF23-centered biomarker discovery: Serial assessment of FGF23, Klotho, and FGFR pathway activity in CKD and AKI cohorts should be integrated with fibrosis-related outcomes to determine whether endocrine FGF abnormalities identify high-risk populations.
2. Mechanistic validation: Cell-specific FGFR3 perturbation and spatial receptor-activation mapping should test the FGF18 and FGF9 pathways across independent renal models before broader mechanistic or therapeutic conclusions are drawn.
3. Therapeutic evaluation: FGF23-directed, Klotho-restorative, broad FGFR, and potential FGFR3-selective strategies should be compared using integrated fibrosis, renal function, and mineral-metabolism endpoints, with therapeutic claims limited to the evidence tier supporting each approach.
4. Clinical guardrails: Oncology FGFR inhibitor success and extra-renal multikinase experience should not be extrapolated directly to CKD fibrosis. Current evidence supports biomarker and mechanistic studies, not clinical FGFR3 blockade.

Renal fibrosis therapy is already being reshaped by SGLT2 inhibitors, nonsteroidal mineralocorticoid receptor antagonists, and other disease-modifying strategies. Any future FGFR3/FGF23-directed approach will therefore need biomarker-defined, mechanism-based benefit beyond current standards of care. FGFR3 should presently be regarded as an emerging ligand-regulated modulator rather than a validated therapeutic target.

### Research Priorities for the Next Five Years

Near-term priorities are:

(i) Spatial mapping of FGFR3/phospho-FGFR3, FGF18, and FGF23/Klotho components in human CKD biopsies.
(ii) Kidney organoid and microfluidic models for FGF23-driven tubular–stromal signaling.
(iii) Genetic perturbation using inducible cell-type-specific FGFR3 perturbation remains an essential experimental prerequisite to disentangle ligand-dependent, cell-type-specific functions of FGFR3 across independent renal injury models for validation of the FGF18- and FGF9- associated signaling pathways.
(iv) Comparative evaluation of Klotho restoration, FGF23-directed approaches, and FGFR modulation using renal and mineral-metabolism endpoints;
(v) Harmonized FGF23 assays and multicenter cohorts integrating endocrine and spatial molecular profiling.

These studies should determine whether FGFR3 can become a biomarker-defined therapeutic target or remains too context-dependent for broad CKD intervention.

## Figures and Tables

**Table 1:** Evidence hierarchy used to interpret FGFR3- and FGF23-related literature in renal fibrosis.

Tier	Definition	Representative Evidence in This Review	Interpretive Use
A	Direct receptor-specific renal experimental evidence	FGF18–FGFR3 and FGF9–FGFR3 studies [17,18]	Supports model-specific renal mechanisms; requires independent replication and human validation.
B	Renal FGF/FGFR evidence without FGFR3-specific attribution	FGF23-driven renal fibrosis; FGF2/FGFR and FGFR1/2 renal studies [19,28,29,30,31,32,33,34,35]	Provides kidney mechanistic context but cannot be assigned to FGFR3.
C	Human renal/CKD associative or biomarker evidence	FGF23/Klotho cohorts, vascular FGFR3 detection, kidney spatial atlases [21,26,27,36,37,38]	Supports clinical relevance or localization, not receptor-specific causality.
D	Extra-renal mechanistic FGFR3 evidence	Systemic sclerosis, pulmonary fibrosis, skeletal and vascular studies [14,15,25,26,36]	Provides biological plausibility; extrapolation to kidney must be explicit.
E	Oncology/skeletal genetics and therapeutic-safety extrapolation	FGFR3-altered urothelial cancer and FGFR inhibitor experience [8,11,16]	Informs receptor pharmacology and safety; does not establish CKD efficacy.

**Table 2:** Representative FGF/FGFR pathway evidence relevant to experimental renal fibrosis.

Intervention/Model	Target/Pathway	Renal Fibrosis Outcome	Species/Model	Ref.
FGF18 augmentation	FGF18-associated FGFR3–AMPK signaling	Attenuated	Folic acid nephropathy, mouse	[17]
Fibroblast FGFR2 knockout	FGFR2	Attenuated	Ischemia/reperfusion fibrosis, mouse	[34]
Endothelial FGFR1 deficiency	FGFR1	Attenuated	Diabetic kidney fibrosis, mouse	[50]
Angiotensin II + FGFR1 pathway modulation	FGFR1	Attenuated	Hypertensive kidney injury, rodent models	[31,32]
FGF2 neutralization/autophagy inhibition	FGF2–FGFR signaling	Attenuated	Post-AKI fibrosis, mouse	[28,29]
Excess FGF23	FGF23-associated FGFR signaling	Increased	Cardiorenal syndrome, mouse	[19]
FGFR3 decoy receptor	FGFR3 ligand trapping	Attenuated (non-renal)	Pulmonary fibrosis	[15]

**Table 3:** FGF23-associated FGFR signaling: renal and cardiorenal evidence.

Domain	Finding	Model/Population	FGFR3 Relevance	Ref.
Physiology	FGF23 signals through FGFR1c/Klotho under physiological conditions; excessive FGF23 may activate alternative FGFR pathways in a Klotho-deficient environment	Experimental/review	No established renal FGFR3 role; context-dependent involvement remains hypothetical	[20,23]
CKD epidemiology	FGF23 rises early during CKD progression and predicts cardiovascular events and renal outcomes	Human cohorts	Indirect	[21,27]
AKI–CKD transition	Elevated FGF23 associated with adverse outcomes after AKI	Clinical cohorts	Indirect	[26]
Direct fibrosis	Excess FGF23 promotes renal fibrosis in experimental cardiorenal syndrome	Mouse model	FGFR subtype unresolved; does not establish FGFR3 causality	[19]
Vasculature	FGF23 and FGFR3 expression detected in arterial tissues associated with impaired kidney function	Human vascular tissue	FGFR3 detected; extra-renal associative evidence only	[36]
Transplant bone	Glucocorticoid-associated FGF23/FGFR3 signaling affects growth plate biology	Pediatric kidney transplant recipients	Functional skeletal FGFR3 signaling	[25]
Klotho axis	Klotho deficiency modifies FGF23 signaling and FGFR pathway balance	Experimental/review	Regulates FGFR utilization	[22,59]
Local paracrine signaling	FGF18–FGFR3 signaling mediates tubular protective responses during renal injury	Mouse renal fibrosis model	Direct renal FGFR3 evidence	[17]

## Data Availability

No new data were created or analyzed in this study. Data sharing is not applicable to this article.

## Abbreviations

AKI, acute kidney injury; CKD, chronic kidney disease; CKD-MBD, chronic kidney disease–mineral and bone disorder; ECM, extracellular matrix; EMT, epithelial–mesenchymal transition; FA, folic acid; FGF, fibroblast growth factor; FGFR, fibroblast growth factor receptor; KIM-1, kidney injury molecule-1; LOX, lysyl oxidase; NGAL, neutrophil gelatinase-associated lipocalin; OBS, sodium p-perfluorous nonenoxybenzenesulfonate; PFAS, per- and polyfluoroalkyl substances; RAAS, renin–angiotensin–aldosterone system; ROS, reactive oxygen species; RTK, receptor tyrosine kinase.
